# Filling Teeth

**Published:** 1873-05

**Authors:** J. Clark Scott

**Affiliations:** Lancaster, O.


### FILLING TEETH.

BY J. CLARK SCOTT, D. D. S., LANCASTER, O.

EDITOR REGISTER :—While I am well aware that under this head a great deal has been written, I am as well convinced that too much can not be written ; for, first, saving the natural organs by successful filling must be acknowledged by all to be the crowning end of the dental art ; and, secondly, every man engaged in the profession knows that a large amount of unsuccessful work is every where done. For these failures there are reasons which can be assigned, but which it is needless to occupy much space in referring to.

I shall assume that good filling and success can be attained, in every case where the normal conditions of the teeth and of the mouth will admit of the attainment ; and therefore I must be allowed to affirm that failures and imperfections under proper conditions, are chargeable alone to the dentist. This affirmation I expect will he acceded to by all.

I may not say much that is new ; but if I say no more than what has often been well said before, I shall hope to be in some degree instrumental in holding the minds of operators to the importance of the work they undertake to do, as well as to remind them of the moral responsibilities they assume.

Starting, then, with the admission that an amount of imperfect tooth filling is every where encountered, we may briefly refer to some of the reasons that will explain.

It is not necessary to say much of the degree of skill and innate ability to successfully prosecute mechanical manipulations that distinguish some men from others, even when their very best efforts have been put forth. There are natural differences in men's abilities in this respect, as well as in every thing else, and which nevertheless involve no moral accountability beyond the fact that, whenever any one becomes innately convinced of this lack of talent as a successful manipulator of the teeth, his duty to himself, and to the public, requires that he should at once abandon the profession and direct his efforts to some calling better suited to his abilities. But of this every one must be the judge for himself.

And neither do I propose to say much of the relative value of the old method of wedge, or ball work—and the adhesive or dry method that can only be accomplished by the employment of the "rubbercloth" and "the mallet." I am satisfied that a great deal of good filling has been done in small round cavities, with or without the use of the mallet; work that has stood the test of years, and that will continue to save the teeth, perhaps through to the end of natural life. But I am fully satisfied that no such success can be reached by the old methods, in the case of proximal and difficult cavities.

To reach excellence in tooth-filling, then, the dentist must possess natural ability, to which must be added a thorough educational ability. These qualifications possessed, the judgment will be called into requisition in every case that is presented, to decide on the diagnosis, and the methods to be adopted, when the whole mind is to be concentrated on the work, both in preparation of cavities and adapting the metal to them, until the work is completed. To know what is to be done, and how to do it, is the whole knowledge required; and he who is thus qualified, and then gives himself earnestly and unremitingly to the case he has in hand, will succeed, while he who is deficient in any of the requirements above specified, will fail.

But no matter what any one's qualifications may be, if he can not command himself; if he can not feel entirely self reliant; not once doubting his ability; or his ultimate success; while at the same time he is so thoroughly self-poised that none of the annoyances so frequent in tooth filling can in the least disturb his equanimity, he will never-the-less fail to accomplish first class work.

A dentist must be so thoroughly himself in all things, as to at least be satisfied with himself. A man who has good judgment, and competency, and who is satisfied with himself, is a safe man to trust in any of the affairs of life. A wrong doer, or one who has defects he can not overcome, is never satisfied with himself, and never makes others satisfied with what he is or does.

These are as much lessons for the dentist to know as anything that concerns him in life.

